# Reply to Lovley, “Untangling Geobacter sulfurreducens Nanowires”

**DOI:** 10.1128/mbio.01041-22

**Published:** 2022-06-01

**Authors:** Xing Liu, Kenneth H. Nealson, Shungui Zhou, Christopher Rensing

**Affiliations:** a Fujian Provincial Key Laboratory of Soil Environmental Health and Regulation, College of Resources and Environment, Fujian Agriculture and Forestry University, Fuzhou, China; b Department of Earth Science, University of Southern California, Los Angeles, California, USA; University of Illinois at Chicago

**Keywords:** bacterial nanowires, *Geobacter*, extracellular electron transfer, anode biofilm, nanowires, pili

## REPLY

Research on Geobacter sulfurreducens nanowires is a hot topic that is full of controversies and unknowns. Cytochrome OmcS and OmcZ nanowires have been detected on G. sulfurreducens growing on an anode by cryo-electron microscopy and are well documented in the literature (1–3). In contrast, due to the lack of structural information for pili, the identification and function of pili remain controversial (4–6). Particularly, a recent study suggested the identity of pili was akin to type II secretion pseudopili in the periplasm, and consequently, the nonexistence of pili on the cell was proposed (5). However, based on our studies (7, 8) and referencing current literature (3, 9–11), we prefer a model having those three nanowires on the surface of G. sulfurreducens growing on an anode.

**FIG 1:**
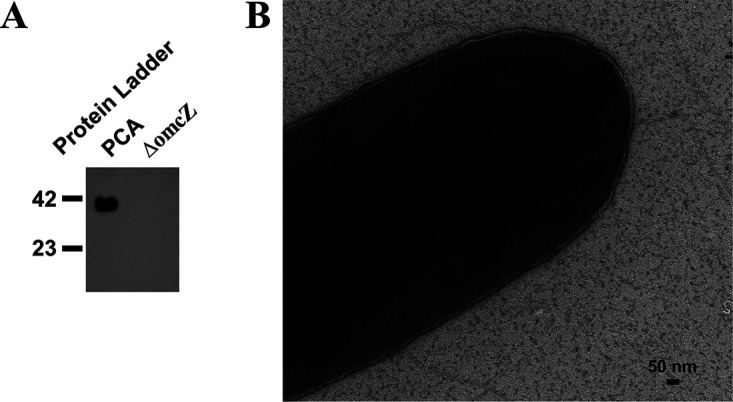
Strain verification. (A) Western blot analysis of cell-free lysate from G. sulfurreducens strain PCA and Δ*omcZ*. (B) Transmission electron microscopy image of strain Δ*pilB* collected from anode biofilm.

**FIG 2:**
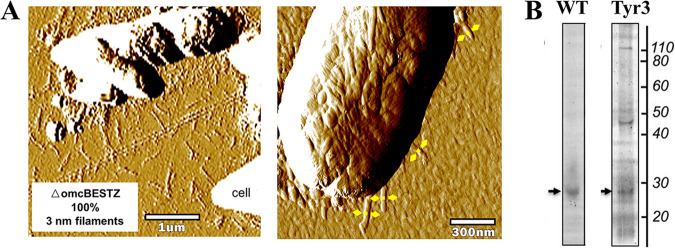
Data from previous studies. (A) Atomic force microscopy images of strain ΔomcBESTZ from (17). (B) Heme-stained proteins were collected from the biofilm matrix of G. sulfurreducens wild-type strain (WT) and strain Tyr3. The gel image was adapted from (14).

In a recent study (12), we investigated the functions of those three nanowires in Geobacter sulfurreducens anode biofilms. To inhibit the expression of an individual nanowire, gene deletions were performed by strictly following the literature. Deletion of *omcS* (13) and *omcZ* (Fig. 1A) was verified by Western blotting, and the deletion of *pilB* was verified by PCR (13). We found a structural role for pili and OmcZ nanowires and conductive roles for OmcS and OmcZ nanowires. In his Letter to the Editor, Lovley questions these conclusions as he alleges our G. sulfurreducens PCA strain only expressed pili. His primary concern lies in the assumption that no filaments were able to be identified in the electron microscopy (EM) figures of our ΔPilB strain. The PilB ATPase is a pilus assembly protein. It has been well documented that a deletion of *pilB* was able to inhibit the expression of pili on the cell but did not affect the secretion of cytochromes (13, 14). Therefore, cytochrome nanowires are expected to be expressed in a Δ*pilB* strain. However, as previous studies have reported, the successful identification of nanowires by EM in G. sulfurreducens was affected by culture conditions and determined by the growth stage (1, 6, 15). In addition, other filaments but not nanowires could also occasionally be identified on G. sulfurreducens (16). Thus, EM examining nanowires is a sophisticated process and deemed not to be a good method to judge the expression and type of nanowires. Instead, atomic force microscopy (AFM) is thought to be a good technique to examine nanowires considering that different nanowires have different surface structures and are assumed to have tiny diameter differences. However, be that as it may, Lovley also barely detected pili on the quintuple cytochrome gene deletion strain ΔBESTZ (Fig. 2A) where he insisted that they were being expressed (17). This recent study also suggested that compared to pili, OmcS nanowires were much less expressed on the cell (17) and OmcZ nanowire expression was stimulated by an electric field (2). Considering our EM samples of a Δ*pilB* strain were collected from an NBAF culture growing at a suboptimal temperature of 25°C which is suitable for the expression of pili, it was foreseeable that the identification of cytochrome nanowires by EM on those cells is only by luck. Therefore, no pili were expressed, and it is not surprising that no cytochrome nanowires have been identified on the randomly selected ΔPilB cells. We further tested Δ*pilB* cells collected from the anode (Fig. 1B). As indicated, cytochrome nanowire-like structures were expressed on Δ*pilB* cells. However, we did not take further steps to examine their identity, as it is not only out of the scope of our study but also a technical challenge at present.

Lovley criticized that we mispresented the heme-staining gel published in a previous study (14) to support our assumption that attempts to reduce the conductivity of pili by replacing aromatic amino acids of pilin with alanine are usually incurred at altering the extracellular cytochrome profile. He asserted that the gel showing that replacing tyrosines in the pilus electron transfer pathway with alanines, generating strain Tyr3, did not affect the extracellular cytochrome profile and is included as Fig. 1D in his Letter. It is mysterious to us that he did not include the control gel running the wild-type sample side by side. As shown in Fig. 2B, under no circumstances did we agree that the mutation did not affect the secretion of extracellular cytochromes. Lovley also cited his studies showing that replacing five aromatic amino acids of pilin with alanines did not affect the secretion of OmcS (18). However, no evidence from those studies showed that the extracellular cytochrome profiles were kept intact after the mutations.

Lovley claimed that some of our results conflicted with previous studies. Previous results demonstrated a heterogeneous distribution of OmcZ in the G. sulfurreducens anode biofilm with a higher density at the anode surface (19). Our model suggests that the OmcZ nanowire both plays a structural role to support the formation of a thick biofilm and acts as the main electron transfer path to facilitate electron transfer in the anode biofilm. In this model, OmcZ nanowires generated by cells either near or distant from the anode will converge at the anode surface. Furthermore, previous studies also showed that cells on the anode surface were tightly packed while cells away from the anode were loosely oriented (19–21). Therefore, a dense OmcZ at the anode surface is warranted. Notably, transcriptomic analyses showed that *omcZ* was evenly transcribed throughout the biofilm (22). Thus, OmcZ should not be confined to the anode surface.

The co-first author Liu previously reported that replacing conductive pili with nonconductive Pseudomonas aeruginosa PAO1 pili in G. sulfurreducens slightly enhanced secretion of OmcS and OmcZ but impaired current generation (23). The study suggested a conductive contribution of pili in the current generation of G. sulfurreducens. However, it is noteworthy to note that Pseudomonas pili showed a high binding tendency on the surface which could affect biofilm formation and then simultaneously impair the current generation. Therefore, the study also highlighted the importance of quantifying the structural function of nanofilaments in electroactive biofilms. In a previous study, we showed that deletion of *pilB*, and strain Δ*pilB*, impaired current generation but did not affect direct interspecies electron transfer (DIET) (13). Moreover, it demonstrated that pili contributed to the current generation of G. sulfurreducens but not diet. However, the role of pili in the current generation is unknown because numerous studies suggested that the electron transfer in G. sulfurreducens biofilm was facilitated by cytochromes but not by pili (24–27). In a recent study, we identified an actual structural role of pili in G. sulfurreducens anode biofilms (12). Notably, by no means did we state “e-pili have a minor role in electron transfer through biofilm,” instead e-pili played a minor structural role compared to the OmcZ nanowire in the anode biofilm. Lovley claimed that the current generation of the same Δ*pilB* strain was different between those two studies. The reason is that the anodes used in those two studies were of different sizes. Even though, the current generation between the wild-type strain and strain Δ*pilB* in those two studies was significantly different and did not conflict with our conclusions.

Finally, Lovley claimed that some of our results were not new and had been reported previously. For example, it had been reported that e-pili played a structural role in noncurrent generation biofilm (28). However, we further found only a structural role of pili in the current generation of a biofilm. The previous study also indicated the importance of OmcZ in conducting electrons at the interface between biofilm and anode (19), and the nonfunction of OmcS in the high-density current generation (29, 30). In contrast, our work suggested that OmcZ nanowires not only mainly facilitated electron transfer in the anode biofilm but also had a structural role to support thick biofilm formation. In addition, OmcS nanowires also facilitated electron transfer in the biofilm but did not have a structural function.

In summary, our study first comprehensively examined the functions of three nanowires in the G. sulfurreducens anode biofilm. All the theoretical frameworks were based on previous studies. One significant discovery of our study is determining the structural contributions of nanowires in the anode biofilm formation which we predict to be an important factor to consider when determining the anode biofilm thickness and the current generation in future studies. As mentioned by Lovley, some key questions have popped up after this study, including what is the main form of existence of OmcS and OmcZ on G. sulfurreducens, free or forming nanowires? What is the mechanism to facilitate the formation of cytochrome nanowires? If pili are conductive, how do they not play a conductive function but only have a structural role in the anode biofilm?
